# Effectiveness of Modified Clear Aligner Attachment Designs on Molar Extrusion: An In Vitro Typodont Study

**DOI:** 10.3390/dj13120551

**Published:** 2025-11-22

**Authors:** Aisha Bin Hussain, Tarek Elshazly, Amar Hassan, Ahmed Ghoneima

**Affiliations:** 1 Department of Orthodontics and Pediatric Dentistry, Hamdan Bin Mohammed College of Dental Medicine (HBMCDM), Mohammed Bin Rashid University of Medicine and Health Sciences (MBRU), Dubai P.O. Box 505055, United Arab Emirates; 2 Oral Technology Department, University Hospital Bonn, 53127 Bonn, Germany; 3 Section of Orthodontics, School of Dentistry, University of California, Los Angeles (UCLA), Los Angeles, CA 90095, USA

**Keywords:** clear aligner therapy, composite attachments, tooth extrusion, upper first molar, buccolingual tipping, 3D printing, in vitro study, CBCT, biomechanics, typodont model

## Abstract

**Background/Objectives**: Clear aligner therapy (CAT) has become a popular, aesthetic, and comfortable alternative to fixed appliances. Advancement of in-house 3D printing has improved accessibility and customization of aligners. However, their effectiveness in achieving certain tooth movements, particularly extrusion, remains uncertain. This study aimed to evaluate the effectiveness of modified aligner designs with different attachment configurations in producing extrusion of the upper first molar using typodont models. **Methods**: An in vitro study was performed with 400 clear aligners fabricated from shape memory polymer (Graphy Tera Harz TC-85DAC) using a Uniz Slash-C LCD 3D printer. Aligners were divided into four groups (*n* = 100 each) based on attachment location: no attachment (G1), buccal (G2), palatal (G3), and combined buccal–palatal (G4). Typodont models were used to simulate clinical conditions. Tooth extrusion, inclination, and angulation were measured using CBCT scans (Veraviewepocs 3D R100) and analyzed with Dolphin 3D imaging software. **Results**: Tooth movement varied by attachment configuration. G1 showed negligible extrusion. G2 and G3 produced significant vertical and angular changes, particularly in cusp extrusion and buccolingual tipping. G4 achieved the most consistent and statistically significant extrusion, with mean values of 0.97 mm (palatal cusp), 0.87 mm (mesiobuccal cusp), 0.72 mm (distobuccal cusp), and 1.62° mesiodistal tipping. The extrusion detected at the mesiobuccal cusp was 0.27, 0.41, 0.95, and 0.87 in G1, G2, G3, and G4, respectively. Buccal-only attachments demonstrated limited effectiveness. **Conclusions**: Attachment placement significantly affects the efficiency of clear aligners in achieving upper first molar extrusion. Combined buccal and palatal attachments provide superior control of extrusion and tipping movements. Customized attachment strategies may enhance clinical outcomes in CAT.

## 1. Introduction

The practice of orthodontics has undergone a significant transformation, evolving from traditional methods to advanced, technology-driven approaches. Its early roots can be traced back to ancient Egyptian practitioners, who used simple materials such as metal bands and catgut, versus the current modern fixed orthodontic treatment, which now offers a wide range of bracket systems. These include metal versus ceramic brackets, zero-prescription versus straight-wire appliances, and passive versus active self-ligation, along with options for customized fabrication. While these systems are typically bonded directly to the teeth, removable alternatives such as clear aligners also serve the same purpose of repositioning teeth to achieve functional and aesthetic alignment [1,2,3].

For patients who prioritize appearance and comfort, clear aligners have rapidly become one of the most popular orthodontic treatment options. The system consists of a series of removable, custom-made transparent trays designed to progressively move teeth into the desired position. Despite their popularity, clear aligners remain limited in their ability to correct certain malocclusions and manage complex orthodontic cases [4,5].

Compared with fixed appliances, achieving precise tooth movements with clear aligners presents greater biomechanical challenges. The orientation of active attachment surfaces plays an important role in generating the force systems required for effective tooth movement. Attachments not only provide directional guidance but also contribute to anchorage control, depending on the intended movement. Several factors affect the efficiency of aligner-based tooth movement, including material properties, aligner thickness, degree of activation, and the design and placement of attachments. Collectively, these variables determine the biomechanical performance of aligners in delivering predictable orthodontic outcomes [5,6,7]. The aim of the current study was to evaluate the effectiveness of a modified clear aligner design in producing orthodontic extrusion using typodont models. Specifically, it investigated how attachment location influences the type of tooth movement (tipping versus bodily movement) and assessed the efficiency of the modified attachment system in facilitating extrusion.

## 2. Materials and Methods

**Figure 1:**
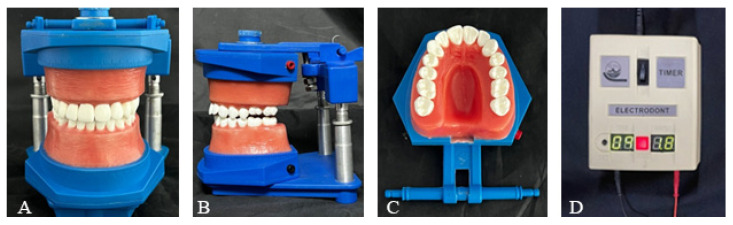
(**A**,**B**) The ElectroDont device used in this study; (**C**) upper arch of the ElectroDont; (**D**) timer and power supply unit.

This in vitro study was conducted using a typodont model (ElectroDonts Savaria-Dent Ltd., Szombathely, Hungary). The typodont was prepared to simulate the extrusion tooth movement (Figure 1). The total study sample consisted of 400 clear aligners, which were divided into four extrusion groups (*n* = 100 per group) based on the location of the attachments. The sample was classified as follows: Group 1 with no attachment, Group 2 with a buccal attachment only, Group 3 with a palatal attachment only, and Group 4 with attachments on both the buccal and palatal surfaces. To ensure objective representation within each group, three distinct sets of aligners were manufactured, each set consisting of ten 3D-printed clear aligners; each aligner was programmed to achieve 0.3 mm of molar extrusion. Extrusion tooth movement was performed 10 times per group: four repetitions were conducted using one set of 3D-printed aligners, while the remaining six repetitions were equally distributed between the other two sets. The sample size was sufficient to detect the observed tooth movement with a power greater than 80% at an α-level of 0.05.

**Figure 2:**
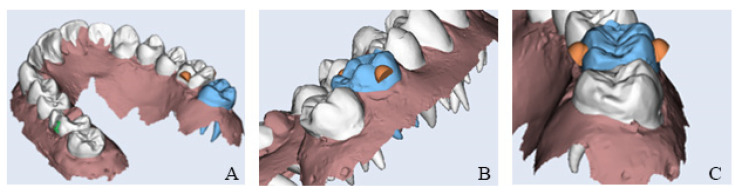
Three-dimensional digital model of the typodont showing the designed composite attachments (orange) and the programmed extrusion of the upper left first molar: (**A**) palatal attachment, (**B**) buccal attachment, and (**C**) attachments on both the buccal and palatal surfaces.

The typodont models were scanned using the RAYIOS2 intraoral scanner (DDS comfort+, Seoul, Korea) to generate digital models in STL format. The models were then imported into Maestro 3D Ortho Studio^®^ software v6 (AGE Solutions^®^, Pontedera, Italy) to design clear aligners with varying attachment configurations on the maxillary left first molar. The optimized attachment was designed with the dimensions of 4 mm in width, 3 mm in height, and 2 mm in depth for all aligners in Groups 2, 3, and 4. A gingival bevel was added to the design to increase surface contact with the aligner and facilitate the extrusion movement (Figure 2). All aligners were fabricated using Graphy Tera Harz TC-85DAC with 100µ shape memory polymer (Graphy Inc., Seoul, Korea) at a standardized thickness of 0.5 mm and printed with the Uniz Slash-C LCD 3D printer (Uniz, San Diego, CA, USA). The aligners were printed using a 100 μm print layer height and positioned in a slanting orientation at 25° to the vertical axis.

**Figure 3:**
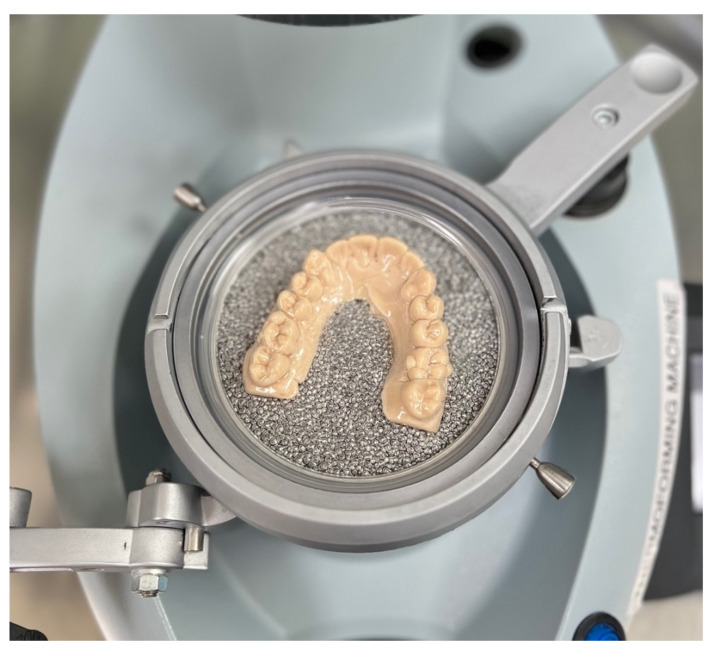
Vacuum-formed retainer with buccal and palatal attachment.

In parallel, a 3D-printed model with the designed attachments was fabricated using Dental Model Z Tan resin. This model served as a template for composite attachment placement on the typodont. A vacuum-formed retainer was produced by applying positive pressure to a heat-softened plastic sheet over the printed mold, replicating the attachment design (Figure 3). Prior to bonding, the maxillary left first molar was mechanically roughened using a round bur in a slow-speed handpiece, cleaned with ethanol and air-dried. A universal primer layer was light-cured for 20 s. Composite attachments were bonded using a light-cure orthodontic composite resin, and polymerization was carried out for 20 s on each surface according to manufacturer instructions [8].

**Figure 4:**
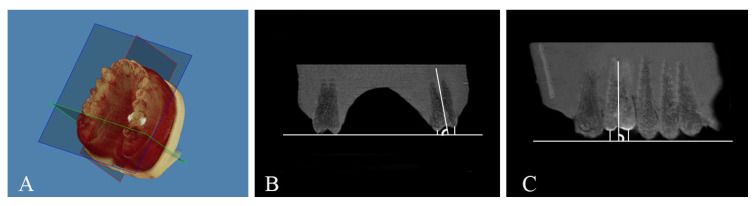
**(A**) Digital model orientation; (**B**) Ext PC, Ext BC, and buccolingual angulation measured from the coronal section; (**C**) Ext MBC, Ext DBC, and MD Tip angle measured from the sagittal section.

**Figure 5:**
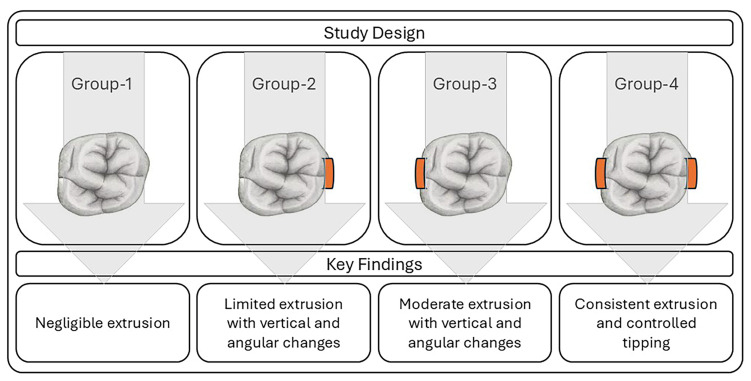
Schematic diagram summarizing the main findings in this study.

Following printing, excess resin was removed using the Tera Harz Spinner centrifuge at 600 rpm for 6 min, with aligners oriented to optimize resin removal. After support removal, aligners were cured in the Cure M machine (Graphy Inc., Seoul, Korea) for 20 min under nitrogen atmosphere to ensure optimal polymerization, then polished with rotating brushes. Cone beam computed tomography (CBCT) scans were taken before the first aligner and after tooth movement (last aligner) using Veraviewepocs 3D R100 (J. Morita Mfg. Corp., Kyoto, Japan) to assess the type of tooth movement achieved by the clear aligners. Tooth inclination, angulation, and vertical displacement were measured using Dolphin 3D imaging software v11.9. Digital models were oriented with the midsagittal plane aligned with the midline of the model, the axial plane represented the occlusal plane by contacting the cusp tips of the last molars and canines, and the transverse plane passed through the mesial aspect of the maxillary second molars, perpendicular to the occlusal plane (Figure 4 and Figure 5 and Table 1).

The ElectroDont device was programmed to follow a controlled thermal cycle consisting of a 10 min heating phase at 50 °C and a 10 min cooling phase. Aligner-0 was placed over the typodont teeth and connected to the power source according to the manufacturer’s instructions. During the heating phase, the softened wax accommodated the programmed extrusion of the upper left first molar. The aligner remained in place throughout the subsequent cooling phase to allow the wax to harden. The typodont was then immersed in cold water for two minutes to ensure complete hardening.

All aligners underwent the same standardized heating–cooling protocol. After each cycle, the aligner was removed, and the device was prepared for the next specimen.

### Statistical Analysis 

To assess measurement reliability, selected parameters were re-measured on 10 randomly selected CBCT scans, with a two-week interval between the initial and second measurements. Intra-rater reliability was evaluated using the intraclass correlation coefficient (ICC). All statistical analyses were conducted using SPSS software version 29.0 (SPSS Inc., Chicago, IL, USA). Continuous variables were summarized using means, standard deviations, and minimum and maximum values for each group. The Shapiro–Wilk test was applied to assess the normality of initial and post-treatment angular and linear measurements, including parameters such as palatal and buccal cusp extrusion. Paired *t*-test was used for comparisons within correlated groups, independent *t*-test for comparisons between two independent groups, and analysis of variance (ANOVA) for comparisons involving more than two group means. A *p*-value of <0.05 was considered statistically significant across all tests. GPower v3.1 was used in this study, and based on detecting a clinically relevant extrusion difference of 0.5 mm with SD 0.5 mm, the sample required *n* = 100 per group for 80% power at α = 0.05.

## 3. Results

Intraclass correlation coefficients were ≥0.90 for all reliability measures. These findings indicate a high degree of measurement consistency between repeated assessments. Descriptive statistics of the initial position of the upper left 1st molar in all groups are presented in Table 2.

The comparison between before and after (T1 − T2) tooth movement was made within each group. Group 1 showed a statistically significant increase in tooth extrusion at mesiobuccal cusp (MBC)—0.27 mm, distobuccal cusp (DBC)—0.25 mm, and BL tips and angle −0.76° with a *p*-value of 0.004, 0.015, and 0.038, respectively. Extrusion of 0.4–0.6 mm was statistically significant in group 2 with a *p*-value of 0.002 for the extrusion of palatal cusp (PC), 0.018 for BC, 0.012 for MBC, and 0.026 for DBC, while the mesiodistal and buccolingual tips were statistically not significant. While for group 3 with palatal attachment, all results were statistically significant; extrusion of around 1 mm was noted in PC, MBC, and DBC and extrusion of 0.5 mm in BC; there was a 3-degree change in the BL tip, causing the tooth to tip buccally, and a −0.6-degree change, causing the tooth to tip mesially. For group 4 with both buccal and palatal attachment, the extrusion of the PC, MBC, and DBC were 0.97 mm, 0.87 mm, and 0.72 mm, respectively, and the MD tip was 1.62 degrees, causing the tooth to tip distally; all were statistically significant. However, the BC extrusion and BL tip were statistically not significant (Table 3).

Table 4, Table 5 and Table 6 represent the comparison between G1 and each of G2, G3, and G4. There was a slight variation in the amount of extrusion in G2 compared to G1, and all measurements were statistically not significant. For G1 vs. G3, the extrusion of PC, MBC, and DBC was statistically significant, with a *p*-value of <0.001, indicating more extrusion was achieved across these parameters with the use of palatal attachment. The BL tip was also statistically significant, with a *p*-value of 0.003, as placement of palatal attachment caused the tooth to tip more buccally, while the MD tip was statistically not significant, with a *p*-value of 0.093. For G1 vs. G4, the extrusion of PC, BC, MBC, and the MD tip was statistically significant; however, the extrusion of DBC and the BL tip was statistically not significant.

## 4. Discussion

The effectiveness of clear aligners in achieving certain types of tooth movement, especially vertical movements such as extrusion, as well as rotational corrections, has always been described as difficult [9,10]. Kravitz et al. [11] identified extrusion as the least predictable movement when using clear aligners. Given the rapid evolution of aligner technology and the current limited source of evidence, accurately predicting the extent and nature of achievable tooth movements remains a challenge. To date, the efficiency of clear aligners in producing specific tooth movements has not been comprehensively assessed or validated. The aim of the current study was to evaluate the effectiveness of a modified clear aligner design with varied attachment locations in producing extrusion of the upper first molar using typodont models.

To enhance the precision and performance of clear aligners, recent advancements have introduced the use of direct 3D printing in their fabrication. This technique offers several advantages, including improved fit, greater flexibility, and favorable viscoelastic properties, which support the continuous application of light forces during orthodontic treatment. Additionally, 3D printing allows for customization of aligner thickness and shape, enabling the design and production of aligners tailored to specific biomechanical requirements [12,13].

The development of 3D-printable biocompatible materials such as Tera Harz TC-85 has been approved by both the European Commission (EC) and the Korea Food and Drug Administration (KFDA), marking a significant advancement in clear aligner technology. Tera Harz TC-85 offers notable clinical advantages, particularly due to its shape memory property, which allows the aligners to exert continuous, consistent orthodontic forces at room temperature without force decay. Additionally, patients can improve comfort and fit by briefly placing the aligners in warm water before use, enhancing flexibility during insertion. Once intraorally warmed to 37 °C, the aligner returns to its original shape and stiffness, even if distorted during placement [13,14,15].

This study initially evaluated how the extrusion of the upper first molar is influenced by the presence or absence of attachments at various sites. In Group 1, without attachments, extrusion at the mesiobuccal and distobuccal cusps measured approximately 0.27 mm and 0.25 mm, respectively; values that were statistically significant but not clinically meaningful due to the insufficient magnitude of movement. These findings align with those of Baik et al. [16], who reported that attachments are essential for achieving effective extrusion, noting that in their study, the absence of attachments led to undesirable intrusion of the lower premolar [16]. Attachments can be classified as optimized or conventional. Optimized attachments are tailored based on tooth morphology and desired movement, while conventional designs include standard shapes such as rectangular or ellipsoidal forms with predefined parameters [17]. In the present study, an optimized attachment (4 mm wide, 3 mm high, and 2 mm deep) with a gingival bevel was used to facilitate extrusion movement.

The extrusion achieved was compared across groups with and without attachments. No significant difference was observed between the no-attachment group (Group 1) and the buccal attachment group (Group 2), suggesting that buccal attachments alone may have limited effectiveness. In contrast, the palatal attachment group (Group 3) exhibited greater extrusion than Group 1. However, this was accompanied by increased buccolingual tipping of approximately 3 degrees in the buccal direction. Although palatal attachments are positioned closer to the tooth’s center of resistance, their biomechanical effects vary depending on the type of movement, highlighting the importance of strategic attachment placement to minimize undesirable tooth tipping [18,19,20].

The group with combined buccal and palatal attachments (Group 4) demonstrated more extrusion than Group 1 and better control over buccolingual tipping, though it did not effectively manage mesiodistal tipping. These findings are consistent with a study by Wang et al., which reported that combined buccal and palatal attachments were most effective in minimizing buccopalatal tipping during second molar intrusion, despite a residual tendency for mesial tipping [21].

When comparing all groups, negative mean differences in extrusion parameters such as the palatal cusp, mesiobuccal cusp, and distobuccal cusp for Group 3 vs. Group 1 indicate greater extrusion. Similarly, a negative mean difference in mesiodistal tipping for Group 4 compared to other groups suggests reduced mesial crown tipping. For buccolingual tipping, Group 3 exhibited the most buccal tipping, followed by Group 4, both of which showed statistically significant differences compared to Groups 1 and 2.

In a study conducted by Groody et al., clear aligners achieved approximately 73% of the predicted extrusion for the upper lateral incisor, with actual movements ranging from 0.3 to 2.5 mm. The degree of extrusion was affected by the type of attachment used, with horizontal attachments yielding comparable outcomes [18]. Similarly, in the present study, the use of attachments, whether placed on the buccal, palatal, or both surfaces, resulted in approximately 0.4 to 1 mm of extrusion at various anatomical landmarks of the tooth, including the mesiobuccal cusp, distobuccal cusp, buccal cusp, and palatal cusp. While some observed differences were statistically significant, their magnitude should be interpreted in the context of clinically meaningful tooth movement.

Typodont models were used in this study to allow direct assessment of the performance of aligners and measurement of actual tooth movement in three dimensions, providing a controlled and reproducible in vitro environment [22,23]. Future studies will integrate the finite element method to complement these findings and further explore biomechanical aspects of tooth movement. This in vitro study on typodont models lacked several clinical variables, such as the periodontal ligament, occlusal forces, and saliva; it was limited to evaluating extrusion of a single tooth, and the wax-based typodont required cooling and stabilization between aligner sequences. Therefore, the findings should be interpreted cautiously when applied to clinical practice.

## 5. Conclusions

This study demonstrated that composite attachments are essential for achieving effective extrusion of the upper first molar with clear aligners. Attachments placed at different locations produced 0.5–1.0 mm of extrusion. Optimal control of buccolingual tipping was achieved when attachments were positioned on both buccal and palatal surfaces, although this configuration did not significantly affect mesiodistal tipping. Palatal-only attachments resulted in greater buccal crown tipping, whereas buccal-only attachments showed minimal differences compared with no attachments.

For clinical application, the use of both buccal and palatal attachments is recommended to maximize extrusion and control buccolingual tipping. Additional auxiliaries may be required to achieve greater extrusion or to manage other undesired tooth movements. However, direct extrapolation to clinical practice should be made with caution, and further in vivo studies are needed to confirm the results.

## Figures and Tables

**Table 1:** Definitions of the selected parameters.

Measurements	Definition
Ext PC	The distance (mm) between the palatal cusp (PC) and the occlusal plane, measured from the coronal section.
Ext BC	The distance (mm) between the buccal cusp (BC) and the occlusal plane, measured from the coronal section.
Ext MBC	The distance (mm) between the mesiobuccal cusp (MBC) and the occlusal plane, measured from the sagittal section.
Ext DBC	The distance (mm) between the distobuccal cusp (DBC) and the occlusal plane, measured from the sagittal section.
BL Tip angle	The buccolingual (BL) angle (degree) between the long axis of the upper left first molar to the occlusal plane, measured from the coronal section.
MD Tip angle	The mesiodistal (MD) tip angle (degree) between the long axis of the upper left first molar to the occlusal plane, measured from the sagittal section.

**Table 2:** Descriptive statistics of the initial position of the upper left 1st molar in all groups.

Group 1	*n*	Mean	SD	Min	Max	95% CI	
						LB	UB
Ext PC—T1	10	1.96	0.30	1.60	2.60	1.75	2.17
Ext BC—T1	10	3.39	0.34	2.90	4.00	3.15	3.63
Ext MBC—T1	10	3.21	0.33	2.90	3.90	2.97	3.45
Ext DBC—T1	10	3.34	0.40	2.80	3.90	3.05	3.63
BL Tip angle—T1	10	73.68	4.12	68.10	81.30	70.73	76.63
MD Tip angle—T1	10	90.63	0.82	89.20	91.70	90.05	91.21
Group 2							
Ext PC—T1	10	2.13	0.38	1.20	2.50	1.86	2.40
Ext BC—T1	10	3.61	0.41	3.20	4.50	3.31	3.91
Ext MBC—T1	10	3.22	0.21	2.90	3.70	3.07	3.37
Ext DBC—T1	10	3.32	0.25	3.00	3.90	3.14	3.50
BL Tip angle—T1	10	73.40	2.85	70.00	78.30	71.36	75.44
MD Tip angle—T1	10	90.47	0.59	89.50	91.40	90.05	90.89
Group 3							
Ext PC—T1	10	2.11	0.27	1.50	2.60	1.92	2.31
Ext BC—T1	10	3.77	0.30	3.40	4.40	3.56	3.98
Ext MBC—T1	10	3.23	0.19	3.00	3.60	3.09	3.37
Ext DBC—T1	10	3.42	0.15	3.10	3.70	3.31	3.53
BL Tip angle—T1	10	70.65	2.60	64.00	72.70	68.79	72.51
MD Tip angle—T1	10	90.98	0.97	90.00	92.50	90.29	91.67
Group 4							
Ext PC—T1	10	2.08	0.26	1.40	2.30	1.89	2.27
Ext BC—T1	10	3.16	0.23	2.80	3.40	3.00	3.32
Ext MBC—T1	10	3.08	0.18	2.80	3.40	2.95	3.21
Ext DBC—T1	10	3.46	0.18	3.20	3.70	3.33	3.59
BL Tip angle—T1	10	72.25	3.25	68.30	77.30	69.92	74.58
MD Tip angle—T1	10	89.27	1.24	86.30	90.90	88.39	90.15

**Table 3:** Comparison between T1 and T2 within each group.

Group 1	T1		T2		Difference		*p*-Value
	Mean	SD	Mean	SD	Mean	SD	
Ext PC	1.96	0.30	1.95	0.25	0.01	0.29	0.916
Ext BC	3.39	0.34	3.18	0.42	0.21	0.35	0.094
Ext MBC	3.21	0.33	2.94	0.40	0.27	0.23	0.004 *
Ext DBC	3.34	0.40	3.09	0.38	0.25	0.26	0.015 *
BL Tip angle	73.68	4.12	72.92	4.23	0.76	0.99	0.038 *
MD Tip angle	90.63	0.82	90.78	0.73	−0.15	0.35	0.213
Group 2							
Ext PC	2.13	0.38	1.52	0.67	0.61	0.46	0.002 *
Ext BC	3.61	0.41	2.98	0.76	0.63	0.69	0.018 *
Ext MBC	3.22	0.21	2.81	0.48	0.41	0.41	0.012 *
Ext DBC	3.32	0.25	2.82	0.61	0.50	0.59	0.026 *
BL Tip angle	73.40	2.85	73.77	3.37	−0.37	2.43	0.642
MD Tip angle	90.47	0.59	90.27	0.83	0.20	0.71	0.399
Group 3							
Ext PC	2.11	0.27	1.12	0.41	0.99	0.53	0.001 *
Ext BC	3.77	0.30	3.25	0.59	0.52	0.47	0.006 *
Ext MBC	3.23	0.19	2.28	0.32	0.95	0.45	0.001 *
Ext DBC	3.42	0.15	2.34	0.42	1.08	0.41	0.001 *
BL Tip angle	70.65	2.60	67.23	3.11	3.42	0.99	0.001 *
MD Tip angle	90.98	0.97	91.58	1.22	−0.60	0.45	0.002 *
Group 4							
Ext PC	2.08	0.26	1.11	0.53	0.97	0.60	0.001 *
Ext BC	3.16	0.23	2.84	0.40	0.32	0.52	0.057
Ext MBC	3.08	0.18	2.21	0.41	0.87	0.50	0.001 *
Ext DBC	3.46	0.18	2.74	0.67	0.72	0.59	0.010 *
BL Tip angle	72.25	3.25	71.07	2.73	1.18	2.34	0.205
MD Tip angle	89.27	1.24	87.65	2.20	1.62	1.41	0.043 *

* Significant at *p* ≤ 0.05.

**Table 4:** Comparison of parameters between Group 1 (G1) and Group 2 (G2) at T2.

Parameters	Groups	T2		Difference		*p*-Value
		Mean	SD	Mean	SD	
Ext PC T2	G1	1.95	0.25	0.01	0.29	0.082
	G2	1.52	0.67	0.61	0.46	
Ext BC T2	G1	3.18	0.42	0.21	0.35	0.477
	G2	2.98	0.76	0.63	0.69	
Ext MBC T2	G1	2.94	0.40	0.27	0.23	0.519
	G2	2.81	0.48	0.41	0.41	
Ext DBC T2	G1	3.09	0.38	0.25	0.26	0.250
	G2	2.82	0.61	0.50	0.59	
BL Tip angle T2	G1	72.92	4.23	0.76	0.99	0.626
	G2	73.77	3.37	−0.37	2.43	
MD Tip angle T2	G1	90.78	0.73	−0.15	0.35	0.162
	G2	90.27	0.83	0.20	0.71	

**Table 5:** Comparison of parameters between Group 1 (G1) and Group 3 (G3) at T2.

Parameters	Groups	T2		Difference		*p*-Value
		Mean	SD	Mean	SD	
Ext PC T2	G1	1.95	0.25	0.01	0.29	0.001 *
	G3	1.12	0.41	0.99	0.53	
Ext BC T2	G1	3.18	0.42	0.21	0.35	0.764
	G3	3.25	0.59	0.52	0.47	
Ext MBC T2	G1	2.94	0.40	0.27	0.23	0.001 *
	G3	2.28	0.32	0.95	0.45	
Ext DBC T2	G1	3.09	0.38	0.25	0.26	0.001 *
	G3	2.34	0.42	1.08	0.41	
BL Tip angle T2	G1	72.92	4.23	0.76	0.99	0.003 *
	G3	67.23	3.11	3.42	0.99	
MD Tip angle T2	G1	90.78	0.73	−0.15	0.35	0.093
	G3	91.58	1.22	−0.60	0.45	

* Significant at *p* ≤ 0.05.

**Table 6:** Comparison of parameters between Group 1 (G1) and Group 4 (G4) at T2.

Parameters	Groups	T2		Difference		*p*-Value
		Mean	SD	Mean	SD	
Ext PC T2	G1	1.95	0.25	0.01	0.29	0.001 *
	G4	1.11	0.53	0.97	0.60	
Ext BC T2	G1	3.18	0.42	0.21	0.35	0.040 *
	G4	2.84	0.40	0.32	0.52	
Ext MBC T2	G1	2.94	0.40	0.27	0.23	0.001 *
	G4	2.21	0.41	0.87	0.50	
Ext DBC T2	G1	3.09	0.38	0.25	0.26	0.085
	G4	2.74	0.67	0.72	0.59	
BL Tip angle T2	G1	72.92	4.23	0.76	0.99	0.131
	G4	71.07	2.73	1.18	2.34	
MD Tip angle T2	G1	90.78	0.73	−0.15	0.35	0.001 *
	G4	87.65	2.20	1.62	1.41	

* Significant at *p* ≤ 0.05.

## Data Availability

The original contributions presented in the study are included in the article, and any further inquiries can be directed to the corresponding author.

## References

[1] Wahl N. (2005). Orthodontics in 3 millennia. Chapter 1: Antiquity to the mid-19th century. Am. J. Orthod. Dentofac. Orthop..

[2] Christensen L.R. (2017). Digital workflows in contemporary orthodontics. APOS Trends Orthod..

[3] Alajmi S., Shaban A., Al-Azemi R. (2020). Comparison of Short-Term Oral Impacts Experienced by Patients Treated with Invisalign or Conventional Fixed Orthodontic Appliances. Med. Princ. Pract..

[4] Galan-Lopez L., Barcia-Gonzalez J., Plasencia E. (2019). A systematic review of the accuracy and efficiency of dental movements with Invisalign^®^. Korean J. Orthod..

[5] AlMogbel A. (2023). Clear Aligner Therapy: Up to date review article. J. Orthod. Sci..

[6] Castroflorio T., Parrini S., Rossini G. (2024). Aligner biomechanics: Where we are now and where we are heading for. J. World Fed. Orthod..

[7] Jedliński M., Mazur M., Greco M., Belfus J., Grocholewicz K., Janiszewska-Olszowska J. (2023). Attachments for the Orthodontic Aligner Treatment-State of the Art—A Comprehensive Systematic Review. Int. J. Environ. Res. Public Health.

[8] Nguyen V.A., Trinh K.L., Le T.L.A., Nguyen H.C. Comparison of shear bond strengths of clear aligner attachments to full-contour zirconia crowns with different sandblasting times and primers: An in vitro study. Am. J. Orthod. Dentofac. Orthop..

[9] Lagravère M.O., Flores-Mir C. (2005). The treatment effects of Invisalign orthodontic aligners: A systematic review. J. Am. Dent. Assoc..

[10] Zheng M., Liu R., Ni Z., Yu Z. (2017). Efficiency, effectiveness and treatment stability of clear aligners: A systematic review and meta-analysis. Orthod. Craniofac. Res..

[11] Kravitz N.D., Kusnoto B., BeGole E., Obrez A., Agran B. (2009). How well does Invisalign work? A prospective clinical study evaluating the efficacy of tooth movement with Invisalign. Am. J. Orthod. Dentofac. Orthop..

[12] Lee S.Y., Kim H., Kim H.-J., Chung C.J., Choi Y.J., Kim S.-J., Cha J.-Y. (2022). Thermo-mechanical properties of 3D printed photocurable shape memory resin for clear aligners. Sci. Rep..

[13] Atta I., Bourauel C., Alkabani Y., Mohamed N., Kim H., Alhotan A., Ghoneima A., Elshazly T. (2024). Physiochemical and mechanical characterisation of orthodontic 3D printed aligner material made of shape memory polymers (4D aligner material). J. Mech. Behav. Biomed. Mater..

[14] Tartaglia G.M., Mapelli A., Maspero C., Santaniello T., Serafin M., Farronato M., Caprioglio A. (2021). Direct 3D Printing of Clear Orthodontic Aligners: Current State and Future Possibilities. Materials.

[15] Narongdej P., Hassanpour M., Alterman N., Rawlins-Buchanan F., Barjasteh E. (2024). Advancements in Clear Aligner Fabrication: A Comprehensive Review of Direct-3D Printing Technologies. Polymers.

[16] Baik J.-C., Choi Y.-K., Cho Y., Baek Y., Kim S.-H., Kim S.-S., Park S.-B., Kim K.B., Kim Y.-I. (2024). Evaluation of different designs of 3D printed clear aligners on mandibular premolar extrusion using force/moment measurement devices and digital image correlation method. Korean J. Orthod..

[17] Hassanaly T., Rabal-Solans A., Mediero-Pérez M., Nieto-Sánchez I. (2024). A comparison of the upper anterior teeth movements with optimized and conventional attachment. J. Clin. Exp. Dent..

[18] Groody J.T., Lindauer S.J., Kravitz N.D., Carrico C.K., Madurantakam P., Shroff B., Darkazanli M., Gardner W.G. (2023). Effect of clear aligner attachment design on extrusion of maxillary lateral incisors: A multicenter, single-blind randomized clinical trial. Am. J. Orthod. Dentofac. Orthop..

[19] Alam M.K., Kanwal B., Shqaidef A., Alswairki H.J., Alfawzan A.A., Alabdullatif A.I., Aalmunif A.N., Aljrewey S.H., Alothman T.A., Shrivastava D. (2023). A Systematic Review and Network Meta-Analysis on the Impact of Various Aligner Materials and Attachments on Orthodontic Tooth Movement. J. Funct. Biomater..

[20] Fan D., Liu H., Yuan C.-Y., Wang S.-Y., Wang P.-L. (2022). Effectiveness of the attachment position in molar intrusion with clear aligners: A finite element study. BMC Oral Health.

[21] Wang Y., Long H., Zhao Z., Bai D., Han X., Wang J., Fang B., Jin Z., He H., Bai Y. (2025). Expert consensus on the clinical strategies for orthodontic treatment with clear aligners. Int. J. Oral Sci..

[22] Elshazly T.M., Keilig L., Alkabani Y., Ghoneima A., Abuzayda M., Talaat S., Bourauel C.P. (2021). Primary Evaluation of Shape Recovery of Orthodontic Aligners Fabricated from Shape Memory Polymer (A Typodont Study). Dent. J..

[23] Elshazly T.M., Keilig L., Alkabani Y., Ghoneima A., Abuzayda M., Talaat W., Talaat S., Bourauel C.P. (2022). Potential Application of 4D Technology in Fabrication of Orthodontic Aligners. Front. Mater..

